# Child mental health problems and poverty

**DOI:** 10.1192/bjb.2024.111

**Published:** 2025-12

**Authors:** Philip Graham, Barbara Maughan

**Affiliations:** 1 University College London, London, UK; 2 King's College London, London, UK

**Keywords:** Child and adolescent psychiatry, mental health services, childhood experience, poverty, attention-deficit hyperactivity disorders

## Abstract

Waiting lists for children and young people with mental health problems are at an all-time high. Almost the only policies proposed to deal with this situation involve increasing the number of mental health professionals. Little attention is given to dealing with the underlying causative stresses, of which poverty is easily the most pervasive. It is suggested that unless levels of poverty are reduced, the rates of psychiatric disorders will not change. As psychiatrists, we need to become much more active in pressing for action over child poverty.

The extent of child mental health problems that have not even been professionally assessed, let alone treated, is perhaps the single most troubling feature of our society today. There are over a quarter of a million children waiting for assessment and treatment, with waiting times of up to 2 years.^1^ The response of government, professional and voluntary organisations is unanimous: the number of mental health professionals must be greatly increased. The need to reduce poverty, the most pervasive stress underlying mental disorders, is barely mentioned. Although, in recent years, the Royal College of Paediatrics and Child Health has pinpointed poverty as a significant stress, none of the other professional or voluntary organisations have followed suit.

To champion an increase in the number of mental health professionals is unexceptionable; the problem is that it is quite unrealistic to suggest that staff levels will rise sufficiently to abolish waiting lists. Worse, provision of early intervention might well result in *higher* prevalence figures and even longer waiting lists. A focus on early identification is likely to result mainly in the recognition of mild problems, many of which would, if left alone, dissipate with time. Further, it is just not rational to intervene therapeutically when the stresses that have produced a mental health problem continue to operate after an intervention has been applied, successfully or unsuccessfully.

## Stresses and adverse events in the young: the role of poverty

So, what are the stresses that result in child and adolescent mental health disorders? The most common adverse influences affecting children are maltreatment of all types, disharmonious family relationships (especially parental conflict and breakdown), academic failure, parental mental illness, brain dysfunction and lack of community support arising from weak neighbourhood cohesion. These are more likely to produce mental health disorders if they are present in combination; most children showing mental health disorders are subject to multiple stresses. However, there is one common factor underlying these stresses and therefore, indirectly, child and adolescent mental health problems – poverty.

## The links between poverty and different types of child mental health problems

From a population perspective, the influence of both absolute and relative poverty varies depending on the type of mental health problem in question. For some conditions, especially eating disorders and autism, poverty is relatively unimportant in the *development* of the problem. This does not mean that socially disadvantaged families with children with these conditions are not additionally burdened by their low income. For example, low-income parents of children with autism will have greater problems accessing appropriate services than those with adequate incomes, but poverty in itself does not act as a cause of autism.

Then there are those mental health problems where poverty is sometimes a major contributing factor and, at other times, a relatively unimportant influence. Most depressive and anxiety disorders fall into this category. For example, the child of a single mother living in cramped accommodation, harassed by debt repayments and finding it difficult or impossible to buy enough food to stave off hunger, will have a high risk of poverty-influenced depression. On the other hand, income is not a stress for children with wealthy parents who have separated acrimoniously with little concern for their wishes or needs, yet such children may well become depressed. Similarly, a child's anxiety state may relate to fear of being sexually abused even in a high-income family. There is one type of anxiety, emotionally based school avoidance, which does seem to be particularly strongly linked to low income. This occurs when children are just too anxious to attend school and parents are unable to get them there. Currently, in the UK, at one time or another, three in ten schoolchildren are missing school for this reason, and the problem is far more common in children from families with a low income.

Finally, there are two psychiatric disorders that are particularly strongly associated with psychosocial disadvantage – conduct disorder and attention-deficit hyperactivity disorder (ADHD) – which, together, account for about half of the total number of mental health disorders in children and young people. Population studies show strong links between low social status and the prevalence of oppositional and conduct disorders.^2^ Children from disadvantaged backgrounds are also far more likely to show ADHD than those from more affluent backgrounds. Thus, in one UK population study,^3^ 15.8% of children from economically inactive families showed ADHD-relevant symptoms compared with 4.3% in managerial and professional families. Poverty affects the most important stresses leading to emotional and behaviour disorders of childhood: child maltreatment, marital disharmony, educational failure and brain dysfunction.^4,5^

## Policies to reduce poverty

We live in a wealthy country, and the decision of whether to lift children out of poverty is a political one. The main justification for alleviating poverty in high-income countries like the UK lies in the fact that it is neither fair nor just that any section of society should go without the basic necessities of life,^6^ but the likely effect on reducing rates of behaviour and emotional problems in childhood is an important additional reason for tackling poverty. There are various ways in which this might be achieved. In theory, the benefit systems should allow parents who are out of work or with low income from employment to receive sufficient funds from the state to keep them out of poverty, however defined. However, in recent years, benefits have simply not kept up with the cost of living.

Aside from the existing system, which relies heavily on the capacity of individuals to access the benefits to which they are clearly entitled, there are two other approaches to reduce levels of poverty. The first, the provision of universal basic services, would require a modification of the present system, involving a much greater attempt to ensure all basic services, including the provision of food and adequate housing, are, like healthcare and education, freely available. The second is for a universal basic income. This would involve all citizens, or sometimes just those who have less than a minimum level of income, regularly receiving an income in the form of an unconditional transfer payment, i.e. without a means test or need to work. This proposal is gradually receiving more support and is being trialled in England, with a monthly payment of £1600.^7^

## Effects of reducing poverty on rates of mental health problems

The effects of alleviating poverty using direct financial assistance on mental health outcomes have been poorly researched in adults and hardly at all in children, but there are a number of encouraging pointers.^8,9^ Particularly encouraging evidence based on sound methodology also comes from Copeland et al,^10^ who found continuing effects on rates of conduct disorder 20 years after a randomly selected group of families had received a significant cash payment.

## Implications of importance of poverty for psychiatrists

If poverty is a powerful influence on the factors that contribute to the development of mental health problems, then mental health professionals need to give attention to its alleviation. They will not be able to relieve poverty themselves, but they need to bring pressure on policy makers who have it in their power to do so. They should not hesitate to bring pressure on local authorities to provide the necessities of life for the children they see. Further, they need to ensure that such families are in receipt of all the benefits to which they are entitled. Their clinical interventions should take into account the social disadvantages that these families are experiencing; for example, by recognising difficulties in accessing services. MRCPsych training programmes should include material relevant to the importance of poverty and poverty reduction. Finally, psychiatrists should support strong poverty reduction programmes such as those undertaken in so-called Marmot places, areas taking a proactive approaching to reducing health inequalities.^11^ A number of influential organisations, including the Resolution Foundation, the Rowntree Foundation, the Institute for Public Policy Research, Changing Realities and Save the Children, are pressing for a major increase in welfare spending to tackle poverty in the UK. The Royal College of Psychiatrists should join them, as the Royal College of Psychiatrists of Wales has already done.

## Data Availability

Data availability is not applicable to this article as no new data were created or analysed in this study.

## About the authors

**Philip Graham,** FRCPsych (Hon), is Emeritus Professor of Child Psychiatry at the Institute of Child Health, University College London, London, UK. **Barbara Maughan,** PhD, is Emeritus Professor of Developmental Epidemiology at the Social, Genetic & Developmental Psychiatry Centre, Institute of Psychology, Psychiatry & Neuroscience, King's College London, London, UK.
